# Editorial: Social and personal skills related to physical education and physical activity

**DOI:** 10.3389/fpsyg.2022.1077005

**Published:** 2022-11-25

**Authors:** Juan De Dios Benítez-Sillero, Gemma María Gea-García, Luis Manuel Martínez-Aranda, Alessandro Quartiroli, Eva M. Romera

**Affiliations:** ^1^Department of Specific Didactis, University of Cordoba, Córdoba, Spain; ^2^Faculty of Sport, Catholic University of Murcia, Murcia, Spain; ^3^Department of Psychology, University of Wisconsin-La Crosse, La Crosse, WI, United States; ^4^School of Sport, Health and Exercise Science, University of Portsmouth, Portsmouth, United Kingdom; ^5^Department of Psychology, University of Cordoba, Córdoba, Spain

**Keywords:** physical education, physical activity, positive development, life skills, youth development

Maintaining an active lifestyle is an effective approach to promoting and supporting physical and psychological health throughout the lifespan. Participation in physical education, sport, exercise and leisure-time physical activity may play an important role in the development of an individual's competence, skills, and social and personal abilities. However, nowadays children and adolescents are immersed in a sedentary society, where healthy lifestyle choices can be influenced by different issues leading to a decrease in the practice of these activities linked to active and healthy lifestyles. As an example, industrialization and increasing technological development have led children and adolescents to acquire increasingly sedentary lifestyles, as well as being exposed to a series of other psychological problems and risks. In this case, a good example could be those related to personality alteration, which enhance certain symptoms such as increased irritability, depression and/or nervousness, among others. Each and every one of these symptoms is harmful to the mental health of this population group. Actually, they are also exposed to other types of situations or alterations such as those related to eating disorders or the acquisition of inappropriate eating habits, as well as altered sleep patterns (Klavina et al.).

This special issue was intended to increase the knowledge of the benefits of engaging in an active lifestyle habit and the practice of well-planned physical education for the improvement of physical and psychological health. Likewise, it also aims to increase the positive evidence of planned interventions, particularly those having a more effective impact on the achievement of some of these physical, educational or psychosocial aspects, through physical education or physical-sporting activity (Figure 1).

**Figure 1:**
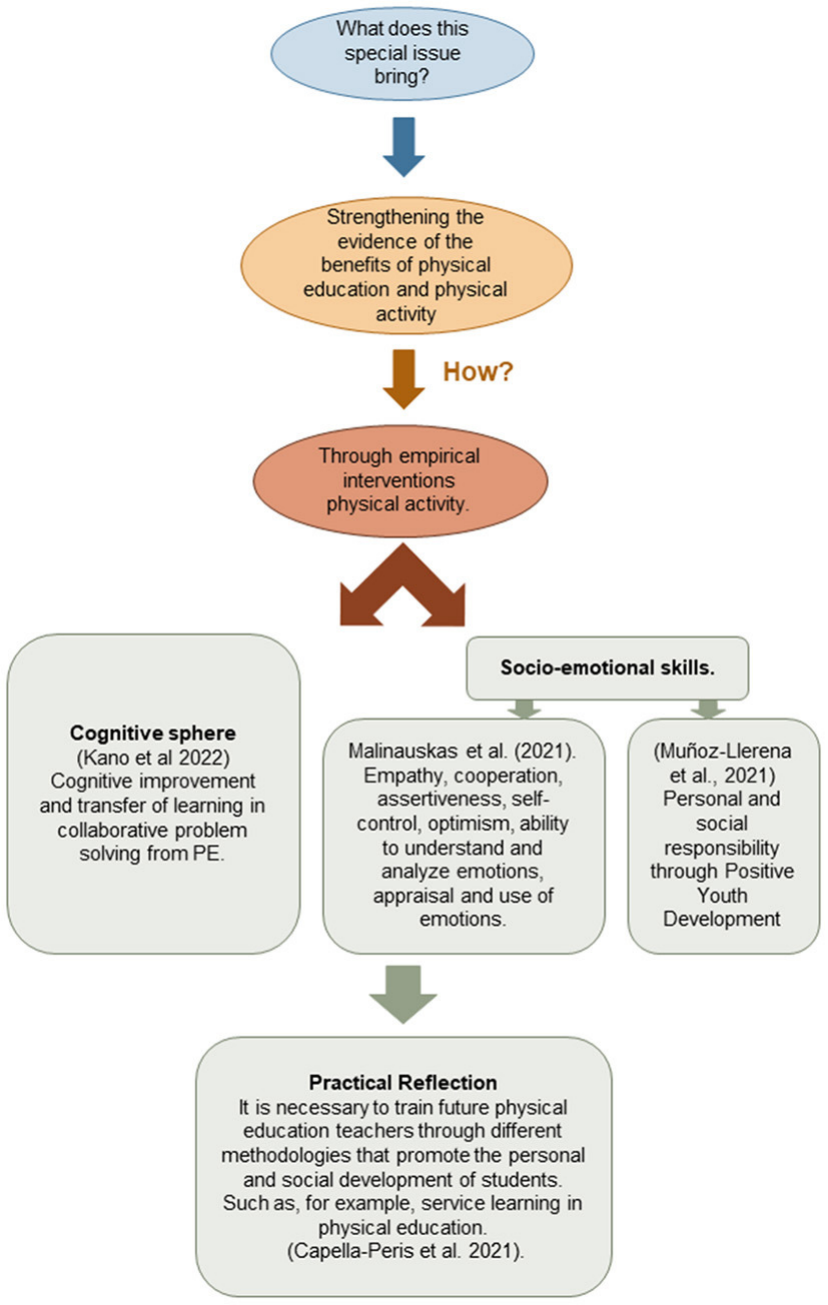
Summary of special issue contributions.

Physical education (PE) subject, it may provide benefits in four related areas: cognitive, affective, psychomotor and social. Interventions in the PE field provide scientific evidence of improvements in these four areas, highlighting the potential of PE not only as a mere instrument for improving the teaching-learning process in the classroom, but also as a tool with great potential for the development of healthier lifestyles in students. This is accomplished by raising awareness of appropriate or unhealthy habits, allowing the practice of physical activity to be contextualized in an appropriate way, generating active patterns in this population group (Dudley et al.). Being more specific, for example, in the cognitive area, the development of collaborative problem solving through specific approaches and styles within PE contributes to improving this competence when problems of the same type are posed, but from the perspective of mathematics. Therefore, this allows us to draw a conclusion such as the apparent existence of transversality, cognitive improvement and transfer of learning in collaborative problem solving from PE (Kano et al.).

It is also possible to propose intervention programmes for improving socio-emotional skills. A good example is the study developed by Malinauskas et al. In this study, 15-min interventions were carried out during 48 sessions. All sessions focused on enhancing the social-emotional skills of: empathy, cooperation, assertiveness, self-control, optimism, ability to understand and analyse emotions, appraisal and use of emotions. For this purpose, training methods were used including impulse control (autogenic training), post-activity discussion of shared experiences, group learning (cooperative learning), role-play scenarios, video viewing and written student worksheets (Malinauskas et al.). Similarly, with the application of other educational interventions, as in the study by Muñoz-Llerena et al., factors also related to socio-emotional skills such as personal and social responsibility were improved through the design of an adapted educational methodology (Positive Youth Development PYD) in an out-of-school context. These types of studies indicate that not only in formal education, but also in areas related to extracurricular sports activities and organized physical activity, it is more than advisable to propose innovative and hybrid proposals such as PYD, in order to obtain the greatest possible benefits in practice.

On the basis of all the above-mentioned evidence, active teachers and university students of physical education should be trained in these methodologies and interventions promoting the training of learners in social and personal aspects. Along these lines, we could also include and introduce methodologies that are currently being used in a multitude of educational and social projects, such as the service learning (SL) methodology, which fosters the personal and civic values of participants (Capella-Peris et al.). This can be considered a good strategy and tool in the pedagogical, human and social training of future PE teachers.

Summarizing the contributions of this special issue, recommendations for coaches when intervening in competitive sport are suggested, as follows: (a) setting two different types of goals, sporting goals and life skills goals; (b) integrating PYD strategies into coaching tasks; (c) using the methodological strategies offered to facilitate the promotion of PYD and life skills learning; (d) involving all players in all roles throughout the season and letting them make their own decisions; and (e) maintaining a balance between sporting outcomes and PYD intervention (Muñoz-Llerena et al.).

Therefore, in this special issue some contributions on the benefits of the practice of physical activity and physical education have been carried out with a formative intention, based on the evidence provided by interventions developed with scientific thoroughness. This should help teachers in their approach to objectives and the design of teaching practices.

## Author's note

This Research Topic of which this editorial is part is based on the X International Conference of Psychology and Education From Neural to Social Network: Wellbeing and Convivencia, 14-18 June 2021, Cordoba, Spain.

## Author contributions

All authors listed have made a substantial, direct, and intellectual contribution to the work and approved it for publication.

## Conflict of interest

The authors declare that the research was conducted in the absence of any commercial or financial relationships that could be construed as a potential conflict of interest.

## Publisher's note

All claims expressed in this article are solely those of the authors and do not necessarily represent those of their affiliated organizations, or those of the publisher, the editors and the reviewers. Any product that may be evaluated in this article, or claim that may be made by its manufacturer, is not guaranteed or endorsed by the publisher.

